# *Veillonella parvula* outer membrane vesicles increase ICAM-1^+^ neutrophils exhibiting elevated NET formation via ROS–PAD4 signaling

**DOI:** 10.3389/fcimb.2025.1540634

**Published:** 2025-06-11

**Authors:** Lina Xu, Yiting Jiang, Xuri Zhao, Jiabao Zhuang, Huiwen Chen, Teng Li, Zhiyan He, Wei Zhou, Zhongchen Song

**Affiliations:** ^1^ Department of Periodontology, Shanghai Ninth People’s Hospital, Shanghai Jiao Tong University School of Medicine, College of Stomatology, Shanghai Jiao Tong University, National Center for Stomatology, National Clinical Research Center for Oral Diseases, Shanghai Key Laboratory of Stomatology, Shanghai Research Institute of Stomatology, Shanghai, China; ^2^ Department of Stomatology, Taizhou Hospital of Zhejiang Province Affiliated to Wenzhou Medical University, Linhai, Zhejiang, China; ^3^ Laboratory of Oral Microbiota and Systemic Disease, Shanghai Ninth People’s Hospital, Shanghai Jiao Tong University School of Medicine, College of Stomatology, Shanghai Jiao Tong University, National Center for Stomatology, National Clinical Research Center for Oral Diseases, Shanghai Key Laboratory of Stomatology, Shanghai, China

**Keywords:** *Veillonella parvula*, outer membrane vesicles, ICAM-1, neutrophils, NETs

## Abstract

**Background:**

*Veillonella parvula* (*V. parvula*), as an anaerobic Gram-negative coccus, produces outer membrane vesicles (OMVs) to promote bacterial colonization and periodontitis progress. Neutrophils are the first immune cells during bacterial stimulation, while neutrophil extracellular traps (NETs) are the early triggers in the progress of periodontitis. However, the interactions between neutrophils and *V. parvula* are still unknown. Therefore, this study investigated the effects and underlying mechanisms of *V. parvula* OMVs on neutrophils.

**Methods:**

Neutrophil migration, apoptosis, and phagocytosis and reactive oxygen species (ROS) production were assessed following stimulation with *V. parvula* OMVs. The differential gene expression in neutrophils was characterized using RNA sequencing. The correlation between intercellular adhesion molecule 1 (ICAM-1) expression and NET formation was verified through flow cytometry and immunofluorescence. The NET formation-associated components, including cell-free DNA, neutrophil elastase, myeloperoxidase, and the PAD4 expression were analyzed. The frequencies of the ICAM-1^+^ neutrophils and NET formation were evaluated in neutrophils pretreated with CYBB or PAD4 inhibitors.

**Results:**

Neutrophils exhibited robust migration, increased apoptosis, and ROS production following exposure to *V. parvula* OMVs. No significant differences were observed in neutrophil phagocytosis. RNA sequencing analysis revealed a significant increase in the expression of *Icam-1* mRNA. And higher ICAM-1^+^ neutrophils in the *V. parvula* OMVs group enhanced the levels of NET formation via activation of ROS-PAD4 signaling pathway.

**Conclusions:**

*V. parvula* OMVs were initially found to increase the population of ICAM-1^+^ neutrophils, which subsequently exhibited elevated NET formation via the ROS–PAD4 signaling pathway. This study elucidates a novel pathogenic mechanism of *V. parvula* OMVs and highlights the potential of targeting ICAM-1^+^ neutrophils as a therapeutic approach for chronic periodontitis.

## Introduction

*Veillonella parvula* (*V. parvula*) is an anaerobic Gram-negative coccus of the normal flora in the intestinal, oral, and respiratory tracts in both animals and humans (Bechon et al., 2020). *V. parvula* has been associated with various inflammatory conditions, including chronic maxillary sinusitis, inflammatory bowel disease, Sjögren’s syndrome, and deep neck infections (Singh et al., 2021; Rojas-Tapias et al., 2022; Guo et al., 2023). Our previous study identified *V. parvula* as a significant contributor to the association between Alzheimer’s disease (AD) and periodontitis (Qiu et al., 2024). Studies have demonstrated that *V. parvula* co-aggregates with diverse dental bacteria, optimizing the microenvironment to support the growth of late periodontopathogens (Yun et al., 2023). Furthermore, *V. parvula* has been shown to induce cytokine production, orchestrating a cascade of destructive events in periodontal enzymes and mediators, ultimately resulting in irreversible hard and soft tissue damage (Galarraga-Vinueza et al., 2017).

*V. parvula* is considered a bridge species due to its virulence factors, including endotoxic lipopolysaccharide (LPS), lipids, and periplasmic proteins. In addition, its outer membrane vesicles (OMVs) act as delivery vehicles for these factors, thereby stimulating microbial growth through metabolic complements (Richards et al., 2022). OMVs can increase the survival rate of bacteria, activate various sentinel cells and induce cytokine release (Zhang et al., 2020). OMVs are currently recognized as a complex mechanism in intracellular and extracellular interactions, molecular effector delivery, nutrient exchange, host cell immune responses, bacterial stimulation, and biofilm formation. *V. parvula* OMVs have been identified as crucial for the adhesion and biofilm formation capacities of this bacterium (Okamura et al., 2021; Du Teil Espina et al., 2022).

In periodontitis, neutrophils are abundantly present within periodontal tissues and the oral cavity (Jiang et al., 2021). During oral bacterial infections, neutrophils serve as the first and the fastest defenders, migrating to inflamed periodontal tissues and the gingival crevice to combat bacterial invasion (Hirschfeld, 2019). Neutrophils from the circulation are attracted by microbial-derived chemotactic and pro-inflammatory factors into the gingiva. They are of great importance in the maintenance of periodontal homeostasis via reducing the microbial load (Wang et al., 2021).

Neutrophils employ several mechanisms to eliminate the invading pathogens and maintain homeostasis and defense. These mechanisms include phagocytosis, degranulation, reactive oxygen species (ROS) production, and neutrophil extracellular trap (NET) formation (Hu et al., 2023). In a periodontal pocket, where neutrophils encounter an overwhelming biofilm, host cells may undergo cell death. Neutrophils may externalize their lethal weaponry through extracellular degranulation to destroy the biofilm (Yadav et al., 2023). The processes of these scavengers are closely interrelated as effective microbial degradation demands ROS release. In addition, NET formation is accompanied by ROS production and degranulation (Li et al., 2020; Tang et al., 2024).

Alterations in the neutrophil phenotypes may influence their activation and functions. Intercellular adhesion molecule 1 (ICAM-1) expression has been observed in neutrophils as a unique neutrophil subpopulation. It has been described that ICAM-1^+^ neutrophils increase the formation of NETs (Zhong et al., 2021). Studies have demonstrated that ICAM-1^+^ neutrophils contribute to significant increases in neutrophil–neutrophil aggregation (Woodfin et al., 2016; Murao et al., 2020). At present, it remains unknown whether ICAM-1^+^ neutrophils play an essential role in the interaction between bacterial attack and neutrophil defense.

Therefore, we investigated the effects of *V. parvula* OMVs on neutrophil migration and apoptosis for the first time. RNA sequencing was used to assess the *Icam-1* mRNA expression in neutrophils. Furthermore, we identified the capacity of ICAM-1^+^ neutrophils to produce elevated levels of NETs through a mechanism dependent on CYBB-derived ROS and PAD4 activity. Collectively, our findings elucidate the role of ICAM-1^+^ neutrophils in response to *V. parvula* OMV stimulation and reveal a novel mechanism in the chronic inflammatory signal cascade.

## Materials and methods

### *V. parvula* OMVs isolation

*V. parvula* cultures were prepared according to previously established protocols (Liu et al., 2020). OMVs were isolated utilizing a standardized methodology using the ExoBacteria OMV Isolation Kit (System Biosciences, Pal Alto, CA, USA) (Micoli and Maclennan, 2020). The bacterial concentration was determined with a spectrophotometer at an optical density of 600 nm. After the initial centrifugation at 6,000 × *g* for 30 min, the supernatant was collected and filtered through a 0.22-μm membrane to ensure complete bacterial cell removal. The filtrate was then transferred into an ultracentrifuge tube and subjected to ultracentrifugation at 100,000 × *g* for 80 min at 4°C, during which grayish-yellow OMVs were deposited on the bottom of the tube. The supernatant was discarded and the pellet resuspended in phosphate-buffered saline (PBS) before being centrifuged again under identical conditions. The purified OMVs were then resuspended in 1 ml pre-cooled sterile PBS, filtered through a 0.22-μm membrane for concentration, aliquoted, and stored at −80°C to prevent freeze–thaw damage. The protein concentrations of the OMV fractions were confirmed using a BCA protein assay kit (Thermo Fisher Scientific, Waltham, MA, USA). The morphology of the *V. parvula* OMVs was assessed using a transmission electron microscope (TEM) (Thermo Fisher Scientific), and the diameter was determined through dynamic light scattering analysis (Malvern Zetasizer Nano ZS90; Malvern Panalytical, Malvern, UK).

### Neutrophil extraction and *V. parvula* OMV stimulation

Neutrophils were extracted from the bone marrow of 8-week-old male C57BL/6J mice, which were obtained from Suzhou Charles River Laboratory Animal Co., Ltd. (SH9H-2020-A220-1; Suzhou, Jiangsu, China). The isolation process employed discontinuous Percoll gradients (GE Healthcare Co., Princeton, NJ, USA), as previously described (Hendricks et al., 2017). C57BL/6J mice were euthanized and the tibias/femurs were isolated. Marrow cavities were flushed with serum-free RPMI medium, filtered through a 70-μm mesh, and centrifuged. The cell pellets were resuspended before layering onto preformed Percoll gradients (78%, 65%, and 55%). After gradient centrifugation at 800 × *g* without braking, the 65%–78% interface was collected and washed and the erythrocytes were lysed. The final pellets were resuspended in supplemented RPMI medium and incubated under standard cell culture conditions.

The viability and purity of the neutrophils were assessed using Wright–Giemsa staining and flow cytometry (FCM) analysis. FCM analysis involved staining the cells with PerCP–cy5.5–CD11b antibody (Ab) (clone 1A8; BioLegend, San Diego, CA, USA) and PE-Ly6G Ab (clone 1A8; BioLegend) prior to any experimental procedures.

For the experimental setup, the neutrophils (5 × 10^5^/well) were seeded for a 0.5-h incubation before the addition of *V. parvula* OMVs (50 μg/ml). Subsequently, the neutrophils were cultured for 4 h under the same conditions. In specific experiments, the neutrophils were pretreated for 1 h with the NET inhibitor GSK484 (HY-100514; MCE, Monmouth Junction, NJ, USA) at 100 nmol/L or the CYBB inhibitor apocynin (HY-N0088, MCE) at 100 μmol/L prior to stimulation with *V. parvula* OMVs. The experimental groups were designated as follows: the control group, the *V. parvula* OMV group, the *V. parvula* OMV+GSK484 group, and the *V. parvula* OMV+apocynin group.

### Neutrophil migration assay

**Figure 1:**
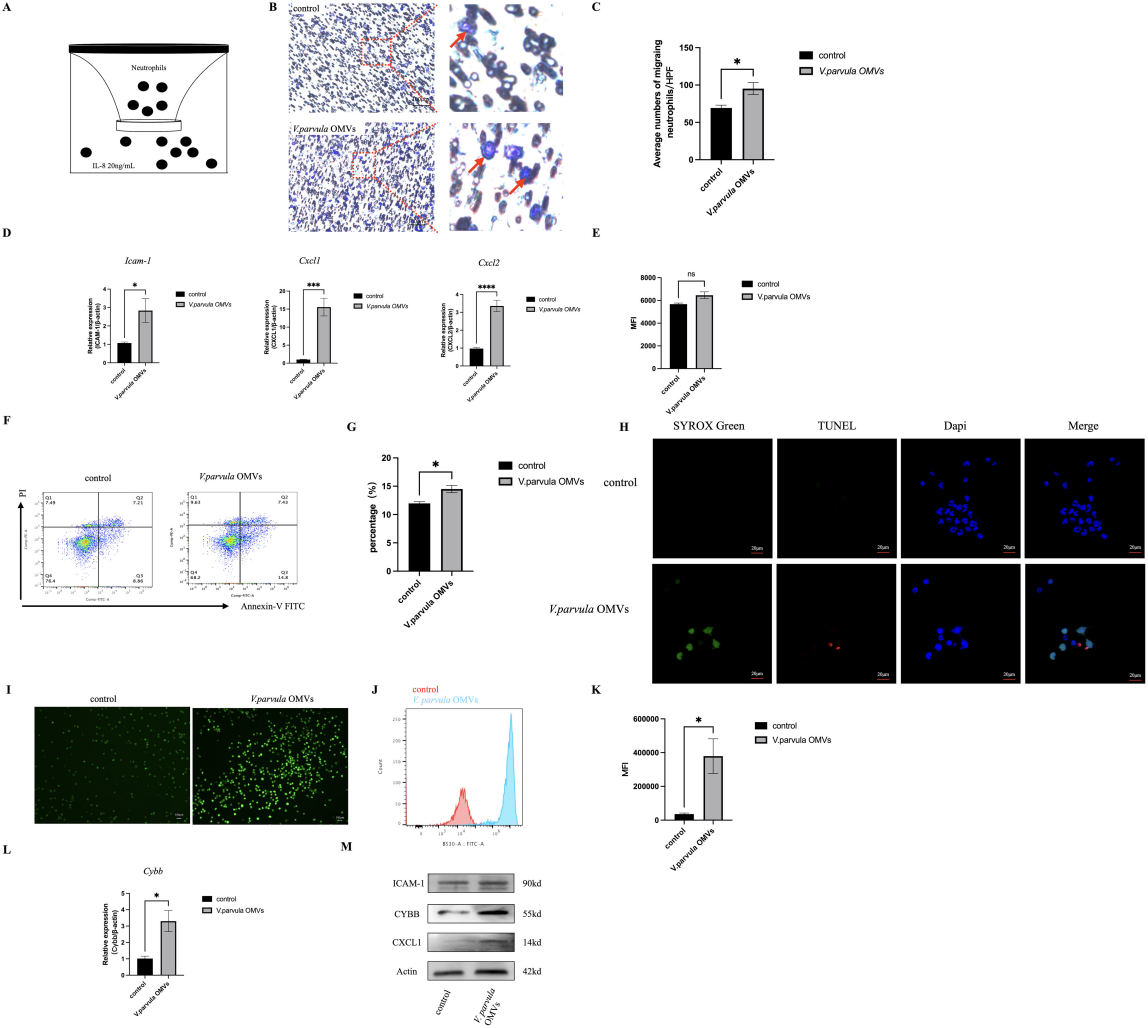
Neutrophil behavior after stimulation with *Veillonella parvula* outer membrane vesicles (OMVs). **(A)** Schematic representation of neutrophil migration *in vitro* for 2 h at 37°C. **(B)** Representative images of migrated neutrophils on the lower membrane stained with 0.1% crystal violet. *Red arrow* indicates migrated neutrophils. *Scale bar*, 100 μm. **(C)** Average numbers of migrated neutrophils per high-power field (HPF; *n* = 6). **(D)** Relative mRNA expression levels of *Icam-1*, *Cxcl1*, and *Cxcl2* analyzed by RT-PCR ***p<0.001, ****p<0.0001 (*n* = 3). **(E)** Mean fluorescence intensity (MFI) of the FITC^+^ neutrophil population in the neutrophil phagocytosis assay ns:Not Significant, p>0.05 (*n* = 3). **(F)** Flow cytometry (FCM) analysis of neutrophil apoptosis. **(G)** Percentage of apoptotic neutrophils (*n* = 3). **(H)** TUNEL (*red*) and SYTOX Green (*green*) staining of neutrophils. TUNEL staining represents neutrophil apoptosis, while SYTOX Green staining represents NETosis. *Scale bar*, 20 μm. **(I)** Reactive oxygen species (ROS) expression in neutrophils. *Scale bar*, 100 μm. **(J)** Histogram of ROS expression. **(K)** MFI of ROS expression (*n* = 3). **(L)** Relative mRNA expression of *Cybb* in neutrophils (*n* = 3). **(M)** Protein expression of ICAM-1, CYBB, and CXCL1 in neutrophils. *p < 0.05 (*n* = 3).

Neutrophils (5 × 10^5^/well) that were either stimulated with *V. parvula* OMVs or left unstimulated for 4 h were then seeded onto the Transwell upper chamber (3 μm; Corning Inc., Corning, NY, USA) for 2 h at 37°C with 5% CO_2_ to evaluate their migration toward the lower chamber. Tissue culture media dissolved with interleukin 8 (IL-8; 20 ng/ml) were added to the basolateral side of each well. The schematic diagram is shown in **Figure 1A**. Following a 2-h incubation period, the non-migratory neutrophils in the upper chamber were cleaned using a small cotton swab. Neutrophils that migrated on the membrane were fixed with a dye fixing solution (Beyotime Co., Shanghai, China) and subsequently stained with a crystal violet staining solution (Beyotime). The resulting images were analyzed using optical microscopy, and the average number of migrated neutrophils per high-power field (HPF) was calculated from six different microscope views.

### Neutrophil phagocytosis assay

All *V. parvula* were labeled with fluorescein isothiocyanate (FITC) to facilitate detection by FCM. The chemical surface labeling process involved culturing bacteria as previously described (Fine et al., 2023), followed by centrifugation and labeling for 1 h at 37°C in 250 µg/ml FITC in an anaerobic chamber.

Neutrophils (1 × 10^5^ cells/ml) that were either stimulated with *V. parvula* OMVs or left unstimulated for 4 h were cultured with a final volume of 100 µl per well. Thereafter, 20 µl of fluorescent bacteria (1 × 10^8^ CFU/ml) was appended to achieve a bacteria-to-cell ratio of 100:1. The reaction mixture was cultured for an additional hour. The mean fluorescence intensity (MFI) of the FITC^+^ neutrophils was recorded. For each sample, 50,000 events were collected. All FCM data were quantified and analyzed using FlowJo version 10.9 software (BD FlowJo, Ashland, Oregon, USA).

### Neutrophil apoptosis assessment

Neutrophil apoptosis was evaluated using FCM. Apoptotic cells were identified using the Annexin V/PI Apoptosis Kit (Beyotime), as described in previous studies (Ou et al., 2022).

The neutrophils were washed three times after *V. parvula* OMV stimulation for 4 h and labeled with 1 ml propidium iodide (PI; 50 mg/L). The PI-stained neutrophils were subsequently tagged with 4 ml Annexin V. FCM analysis counted 10,000 events to determine the number of apoptotic cells. The data obtained from FCM were analyzed using FlowJo version 10.9 software (BD FlowJo).

### TUNEL and SYTOX Green staining

Neutrophils (2 × 10^5^ cells/ml) that were either stimulated with *V. parvula* OMVs or left unstimulated for 4 h were assessed using a TUNEL kit (40308ES20; Yeasen Biotech, Shanghai, China) and SYTOX Green (KGA261; KeyGEN BioTECH, Nanjing, China) following the manufacturers’ instructions. The neutrophils were fixed with a dye fixing solution, treated with proteinase K to reactivate antigens, incubated with an equilibrate buffer, and then labeled with the TUNEL detection cocktail. Afterward, SYTOX Green and DAPI (Beyotime) were applied. After staining for 15 min, TUNEL and SYTOX Green staining was observed under Zeiss LSM880.

### Reactive oxygen species production

Following stimulation for 4 h, neutrophils (2 × 10^5^ cells/ml) that were either stimulated with *V. parvula* OMVs or left unstimulated were incubated with the ROS probe DCFH-DA (10 μmol/L; Beyotime) for 20 min. Subsequently, the neutrophils were washed three times with serum-free RPMI medium to thoroughly remove the DCFH-DA that had not entered the cells. The expression of ROS in neutrophils was directly observed using a Leica Microsystems microscope and analyzed with the LAS X software (Leica, Wetzlar, Germany). The neutrophils were pipetted to dislodge them from the surface, collected, and analyzed by FCM. The MFI was subsequently determined using FCM. The neutrophils in the *V. parvula* OMVs+GSK484 and *V. parvula* OMVs+apocynin groups were also incubated with the ROS probe DCFH-DA.

### RNA sequencing and data analysis

RNA sequencing was evaluated in neutrophils with or without *V. parvula* OMVs stimulation for 4 h by Majorbio Bio-Pharm Technology (Shanghai, China). The total neutrophil RNA was derived using the TRIzol reagent (Takara, Kusatsu, Shiga, Japan). Fragments per kilobase of exon per million mapped (FPKM) were applied to normalize the gene expression levels. The FPKM data were used to elucidate the relationship between the control group and the *V. parvula* OMVs group using principal component analysis (PCA) and hierarchical clustering analysis. Changes in the differentially expressed genes (DEGs) were identified using edgeR with the general filtering criteria of |log2 fold change| > 1.5 and *p* < 0.05 applied to the FPKM values. The DEGs were enriched using Gene Ontology (GO) and Kyoto Encyclopedia of Genes and Genomes (KEGG) annotations to identify the relevant pathways.

### Reverse transcription polymerase chain reaction

The neutrophil total RNA was extracted using the TRIzol reagent. The concentration and the purity of the extracted RNA were assessed to ensure quality. Complementary DNA (cDNA) was synthesized using the PrimeScript RT Master Mix (Takara) according to the manufacturer’s instructions. Reverse transcription polymerase chain reaction (RT-PCR) assays were performed by amplifying each cDNA sample with SYBR Premix Ex Taq (Yesen, Shanghai, China) on an ABI Real-Time PCR 7300 System (Roche, Basel, Switzerland). The relative target gene expression levels were normalized to that of *β-actin.* The primer sequences are presented in **Table 1**.

**Table 1:** Primer sequences of the genes for RT-PCR.

Gene	Primer sequences (F: forward; R: reverse)	Product size (bp)
*Icam-1*	F: ATGCCCAGACATCTGTGTCC	112
	R: GGGGTCTCTATGCCCAACAA	
*Cxcl1*	F: ACTGCACCCAAACCGAAGTC	114
	R: TGGGGACACCTTTTAGCATCTT	
*Cxcl2*	F: CCAACCACCAGGCTACAGG	108
	R: GCGTCACACTCAAGCTCTG	
*Pad4*	F: TGGTCCTCCAGTCAAGAAGAG	84
	R: GCTTTCACCTGTAGGGTCACC	
*Cybb*	F: AGTGCGTGTTGCTCGACAA	106
	R: GCGGTGTGCAGTGCTATCAT	
*β-actin*	F: GTGCTATGTTGCTCTAGACTTCG	174
	R: ATGCCACAGGATTCCATACC	

### Western blot analysis

Protein was extracted and was measured using a BCA protein assay kit. Equal cellular protein was subjected to SDS-PAGE (10%) and then transferred into polyvinylidene fluoride (PVDF) membranes (Millipore, Billerica, MA, USA). After blocking with 5% non-fat dry milk for 1 h as previously described (Qiu et al., 2021), the membranes were incubated overnight at 4°C with primary antibodies. Finally, the membranes were incubated with the corresponding horseradish peroxidase (HRP)-linked secondary antibodies and then scanned using an enhanced chemiluminescence reagent (Thermo Fisher Scientific).

### ICAM-1 or myeloperoxidase expression in neutrophils

To assess the ICAM-1 or the myeloperoxidase (MPO) expression in neutrophils, 1 × 10^6^ neutrophils with or without *V. parvula* OMVs stimulation for 4 h were washed and stained with the PerCP–cy5.5 anti-mouse–CD11b antibody, the PE anti-mouse Ly-6G antibody, the anti-rabbit ICAM-1 antibody (CD54; Proteintech, Wuhan, China), and/or the MPO antibody (HA601249; HUABIO Co., China; Cell Signaling Technology, Danvers, MA, USA). The neutrophils were subsequently incubated with fluorescent-labeled secondary antibodies. Unstained cells served as negative controls, and single-color positive controls were made respectively. FCM data were determined on 60,000 events and were analyzed using FlowJo version 10.9 software. The ICAM-1- or MPO-expressing neutrophils were determined as CD11b^+^Ly6G^+^ICAM-1^+^ or CD11b^+^Ly6G^+^MPO^+^ populations, respectively.

### Immunofluorescence staining

Neutrophils with or without *V. parvula* OMVw stimulation for 4 h were fixed, permeabilized for 15 min, and blocked using an immunostaining blocking solution (Beyotime). Subsequently, the neutrophils were stained with the anti-rabbit ICAM-1 antibody and the anti-goat MPO antibody (R&D Systems, Minneapolis, MN, USA) at 4°C overnight. Coverslips were mounted onto glass slides using an anti-fluorescence quencher containing DAPI (Beyotime) after incubation with the secondary antibodies with fluorescent labeling for 1 h. The images were observed using Zeiss LSM880.

The morphology of the NETs induced was visualized by incubating the cells with the primary anti-goat MPO antibody and the anti-rabbit citrullinated H3 antibody (CitH3; ab5103, Abcam, Waltham, MA, USA). The images were observed using Zeiss LSM880.

### Circulating NET levels

To identify NETs, the circulating cell-free DNA (cf-DNA), neutrophil elastase (NE), and MPO were quantified in the supernatant, as previously described (Wang et al., 2023). Following *V. parvula* OMVs stimulation of the neutrophils for 4 h, the supernatant fractions were collected into 1.5-ml tubes. The supernatant tubes were centrifuged at 450 × *g* for 5 min at 4°C to pellet any detached neutrophils. The supernatant was then transferred to another 1.5-ml tube. Quantification of cf-DNA was performed with the Quant-iT PicoGreen ds-DNA Quantification Kit (Invitrogen, Carlsbad, CA, USA). The NE concentrations were determined using an NE ELISA kit (Yinxing Lab, Shanghai, China). MPO activity was assessed with an MPO Detection Kit (Nanjing Jiancheng Bioengineering Institute, Nanjing, China).

### PAD4 expression

After stimulation of the neutrophils with *V. parvula* OMVs for 4 h, the supernatant fractions were quantified using a PAD4-specific ELISA Kit (Cusabio, Wuhan, China) in strict accordance with the manufacturer’s instructions. The detailed procedure was as follows: 100 μl of the standard product and the sample were respectively added into the preset standard well and the sample well and then incubated at 37°C for 2 h. Once all the liquid had been aspirated, biotin antibody (1×) was added and incubated at 37°C for 1 h. Subsequently, the wells were washed twice and incubated with HRP–avidin (1×) at 37°C for 1 h. The wells were then washed five times and 90 μl of the TMB substrate was added to each well. The wells were incubated in the dark at 37°C for 15–30 min. Subsequently, 50 μl of the stop solution was added to each well. Finally, the optical density of each well was measured within 5 min using a microplate reader set at 450 nm.

### Statistical analysis

Experimental data were expressed as the mean ± standard error and analyzed using GraphPad Prism software (GraphPad Software, San Diego, CA, USA). For comparison between two groups, Student’s *t*-test was used. For comparison of more than three groups, one-way analysis of variance (ANOVA) was employed. Values of *p < 0.05, **p < 0.01, ***p < 0.001, and ****p < 0.0001 were defined as statistically significant.

## Results

### *V. parvula* OMVs characterization and neutrophil purity

**Figure 2:**
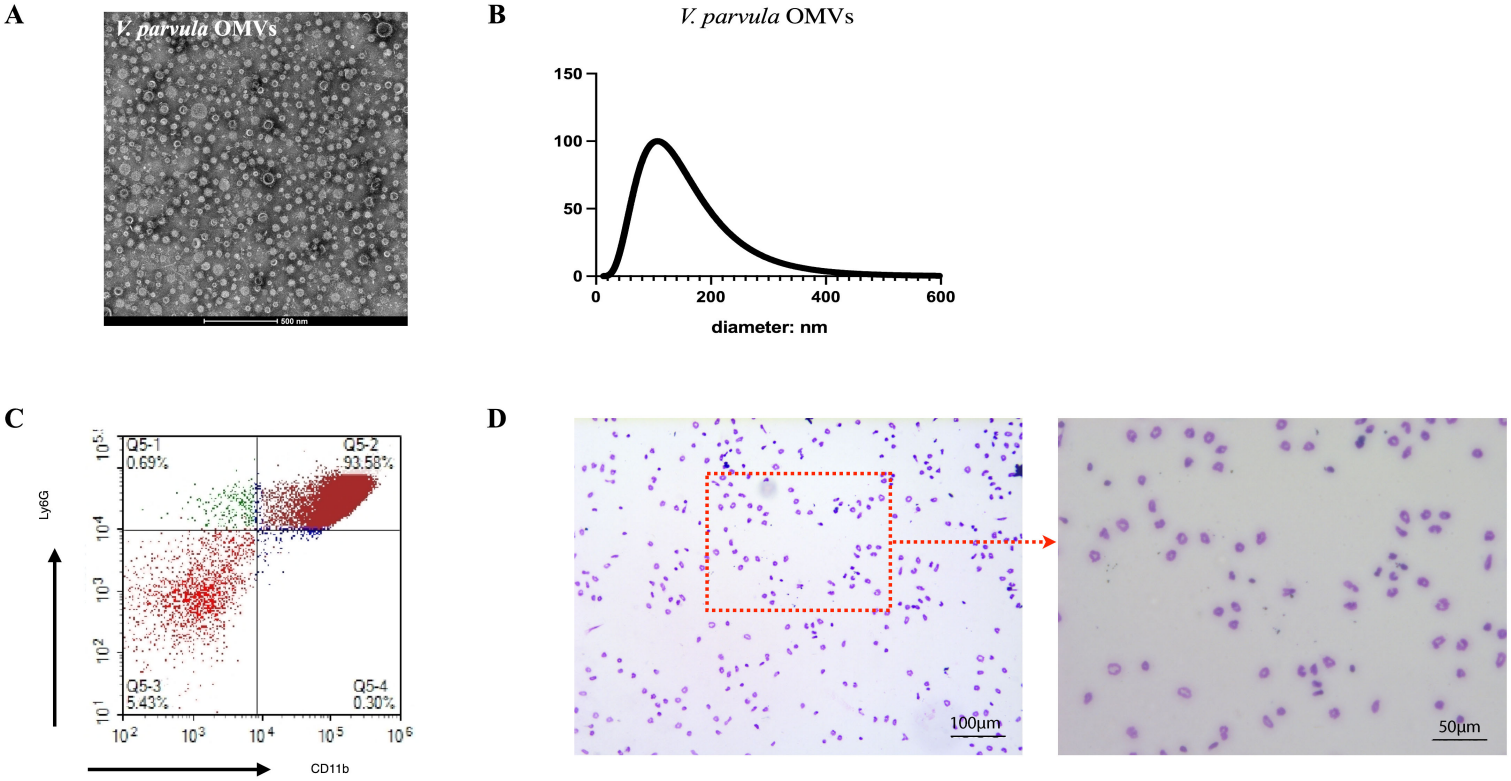
Extraction of *Veillonella parvula* (*V. parvula*) outer membrane vesicles (OMVs) and neutrophils from mouse bone marrow. **(A)** Transmission electron microscopy (TEM) image of *V. parvula* OMVs. *Scale bar*, 500 nm. **(B)** Nanoparticle tracking analysis (NTA) of *V. parvula* OMVs. **(C)** Flow cytometry analysis of the percentage of CD11b^+^Ly6G^+^ neutrophils. **(D)** Giemsa staining of sorted neutrophils. scale bar: 100μm and 50μm.

The *V. parvula* OMVs showed spherical, bilayer cell portions in TEM, which revealed vesicle-like structures (**Figure 2A**). The nanoparticle tracking analysis (NTA) results demonstrated that the average diameter of the *V. parvula* OMVs was 125.7 ± 5.6 nm, similar to the typical OMVs diameters of other Gram-negative bacteria (**Figure 2B**).

The FCM analysis confirmed that the purity of the CD11b^+^Ly6G^+^ neutrophils after Percoll gradient separation exceeded 90% (**Figure 2C**). The Wright–Giemsa staining results revealed the neutrophil morphology, which showed segmentation in the nuclei (**Figure 2D**).

### Neutrophil behavior after *V. parvula* OMVs stimulation

Neutrophils exhibited robust migration in response to *V. parvula* OMVs stimulation (**Figure 1B**, *red arrow* indicating migrated neutrophils). The number of migrating neutrophils increased significantly in the *V. parvula* OMVs group (**Figure 1C**). We also examined whether the *V. parvula* OMVs have an effect on the expression of chemokines. The results showed that the mRNA expression levels of *Cxcl1*, *Cxcl2*, and *Icam-1* were elevated in the *V. parvula* OMVs group (**Figure 1D**). The protein expression levels of CXCL1 and ICAM-1 in the neutrophils of the *V. parvula* OMVs group were notably increased (**Figure 1M**).

The phagocytosis analysis was based on the MFI of the FITC^+^ neutrophil population. The results revealed no significant changes in the *V. parvula* OMVs group (**Figure 1E**).

The frequency of neutrophil apoptosis was significantly increased in the *V. parvula* OMVs group compared with the control group (**Figures 1F, G**), indicating that neutrophil apoptosis was extremely enhanced by the *V. parvula* OMVs. In the control group, the neutrophils exhibited an intact nuclei (DAPI, blue). In the *V. parvula* OMVs group, apoptotic cells (red) showed cell shrinkage and membrane blebbing, while NETotic cells (green) displayed chromatin decondensation, cell swelling, and membrane rupture (**Figure 1H**).

### Neutrophil ROS production

Neutrophils exhibited significantly enhanced ROS levels in the *V. parvula* OMVs group (**Figure 1I**). The *V. parvula* OMVs elicited higher ROS production in neutrophils (**Figure 1J**). The FCM analysis revealed a mean fluorescence intensity (MFI) of 343,007 ± 102,664 relative fluorescence units (RFU) in the *V. parvula* OMVs group, which was substantially higher than the 36,081 ± 2,125 RFU of the control group (**Figure 1K**).

The *Cybb* mRNA expression level was increased significantly in the *V. parvula* OMVs group (**Figure 1L**). Correspondingly, the CYBB protein expression in the neutrophils of the *V. parvula* OMVs group was notably elevated, consistent with the observed mRNA expression patterns (**Figure 1M**).

### RNA sequencing analysis

**Figure 3:**
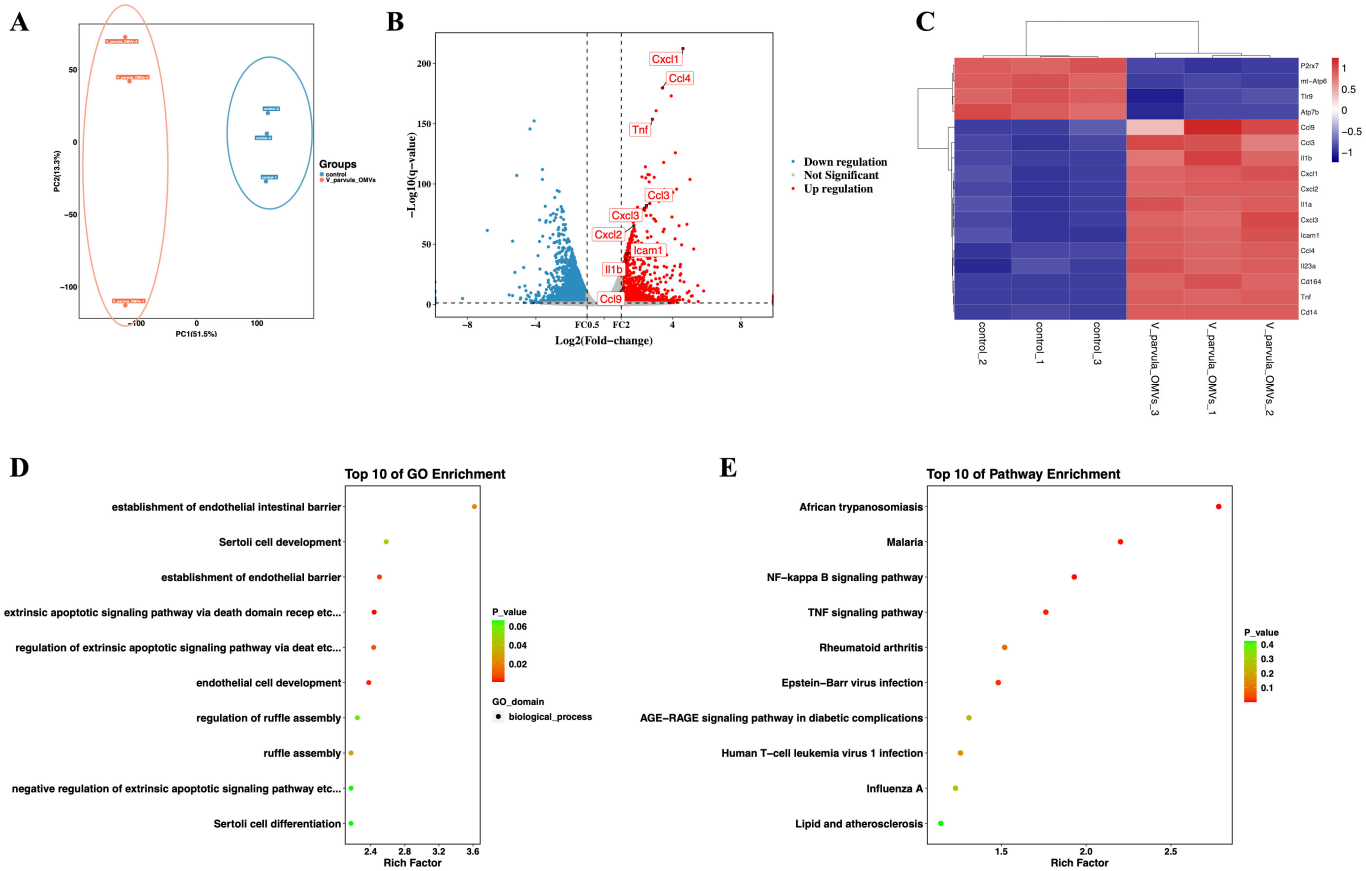
RNA sequencing analysis of neutrophils stimulated with *Veillonella parvula* outer membrane vesicles (OMVs). **(A)** Principal component analysis (PCA) plot. **(B)** Volcano plot depicting the differentially expressed genes. **(C)** Heatmap illustrating the differentially expressed genes. **(D)** Top 10 most significant Gene Ontology (GO) terms in the GO analysis of *Icam-1* expression. **(E)** Top 10 most significant Kyoto Encyclopedia of Genes and Genomes (KEGG) pathways in the KEGG analysis of the *Icam-1* gene (*n* = 3).

To elucidate the interaction between the *V. parvula* OMVs and the neutrophils, RNA sequencing was performed. The PCA demonstrated consistency in the data (**Figure 3A**). The DEGs with increased expression were enriched in inflammatory processes (e.g., *Il-1β* and *Tnf*) and neutrophil chemotactic responses to stimulation (e.g., *Cxcl1*, *Cxcl2*, and *Ccl*3). The volcano plot revealed 640 upregulated and 2,148 downregulated mRNAs in the neutrophils of the *V. parvula* OMVs group compared with the control group (**Figure 3B**). In particular, the *Icam-1* mRNA expression in neutrophils was significantly enhanced in the *V. parvula* OMVs group. A heatmap illustrating the differential gene expression patterns was generated (**Figure 3C**). KEGG and GO pathway enrichment analyses of the *Icam-1* gene expression were conducted. The top 10 most significantly enriched GO terms indicated that *Icam-1* expression is associated with extrinsic apoptotic pathways (**Figure 3D**). The top 10 most significantly enriched KEGG pathways (**Figure 3E**) included TNF signaling and various infection-related pathways.

### ICAM-1 and MPO expression in neutrophils

Both the mRNA and protein levels of ICAM-1 in the neutrophils of the *V. parvula* OMVs group were markedly upregulated (**Figures 1D, M**).

**Figure 4:**
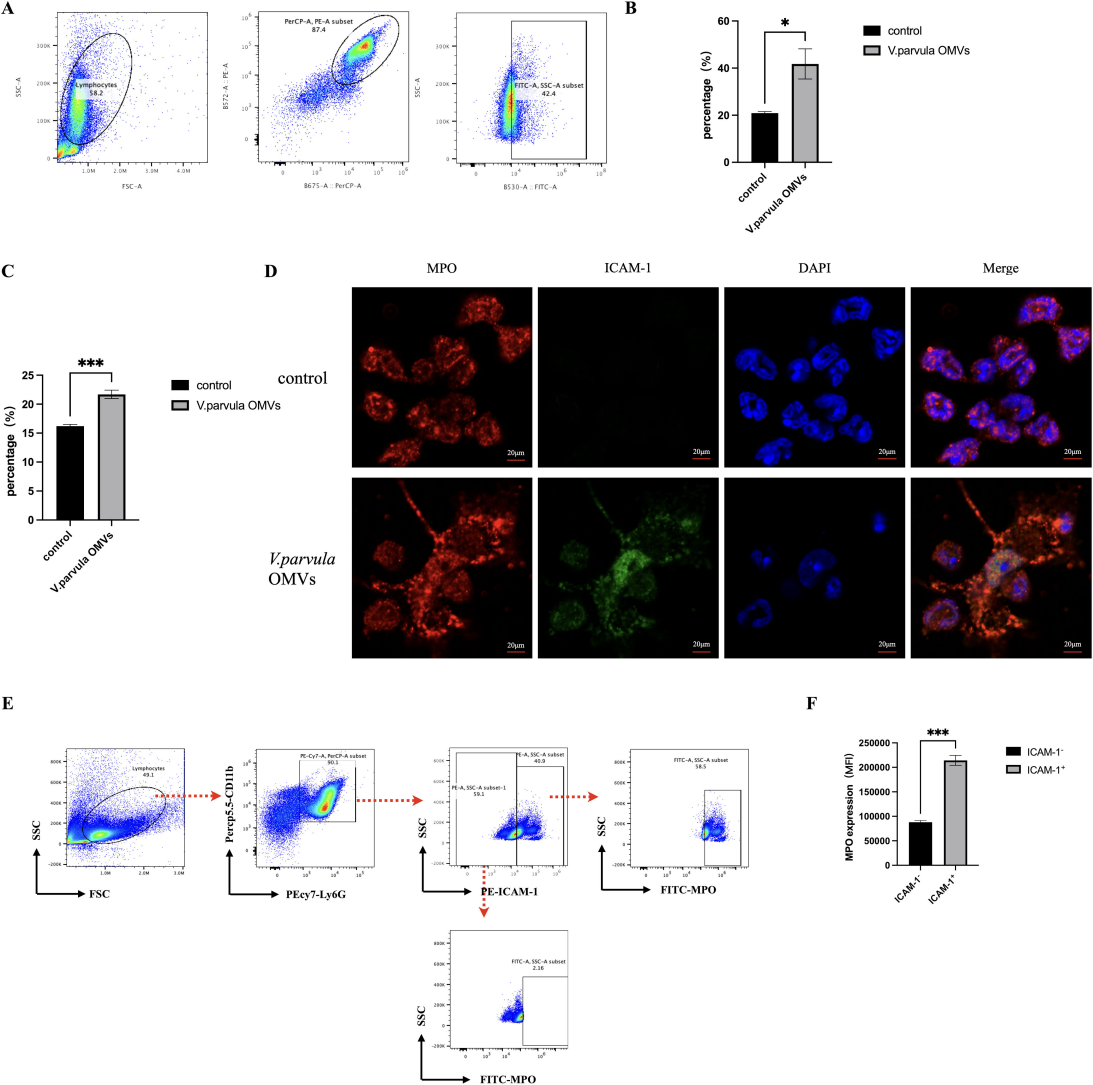
ICAM-1 and myeloperoxidase (MPO) expression in neutrophils after stimulation with *Veillonella parvula* outer membrane vesicles (OMVs). **(A)** Scatter plots of the ICAM-1 expression in neutrophils. **(B)** Percentage of ICAM-1^+^ neutrophils. **(C)** Percentage of MPO-expressing neutrophils. **(D)** Immunofluorescence staining of MPO and ICAM-1 in neutrophils. *Scale bar*, 20 μm. **(E)** Scatter plots representing the frequencies of extracellular MPO in ICAM-1-expressing neutrophils. **(F)** Corresponding mean fluorescence intensity (MFI) MPO expression in ICAM-1^+^ neutrophils compared with ICAM-1^−^ neutrophils. *p < 0.05, ***p < 0.001 (*n* = 3).

FCM analysis of the ICAM-1 expression in neutrophils was performed. The gating strategy is shown in **Figure 4A**. A notable increase in ICAM-1^+^ neutrophils was observed in the *V. parvula* OMVs group (**Figure 4B**). To characterize the ICAM-1^+^ neutrophils in terms of their NET formation capacity, the expression of MPO was analyzed in the *V. parvula* OMVs group using FCM. Stimulation with *V. parvula* OMVs resulted in significantly higher MPO expression levels (**Figure 4C**). Fluorescence microscopy revealed the higher expression of MPO and ICAM-1 in the neutrophils of the *V. parvula* OMVs group than that in the control group. (**Figure 4D**). These findings indicate that ICAM-1^+^ neutrophils exhibit enhanced NET formation. The gating strategy for the FCM analysis of the MPO expression in ICAM-1^+^ neutrophils is shown in **Figure 4E**. It was demonstrated that ICAM-1^+^ neutrophils had significantly higher levels of MPO expression compared with ICAM-1^−^ neutrophils (**Figure 4F**). Surface-bound extracellular MPO serves as a marker for NETs, suggesting that ICAM-1^+^ neutrophils improved NET formation.

### NET formation

**Figure 5:**
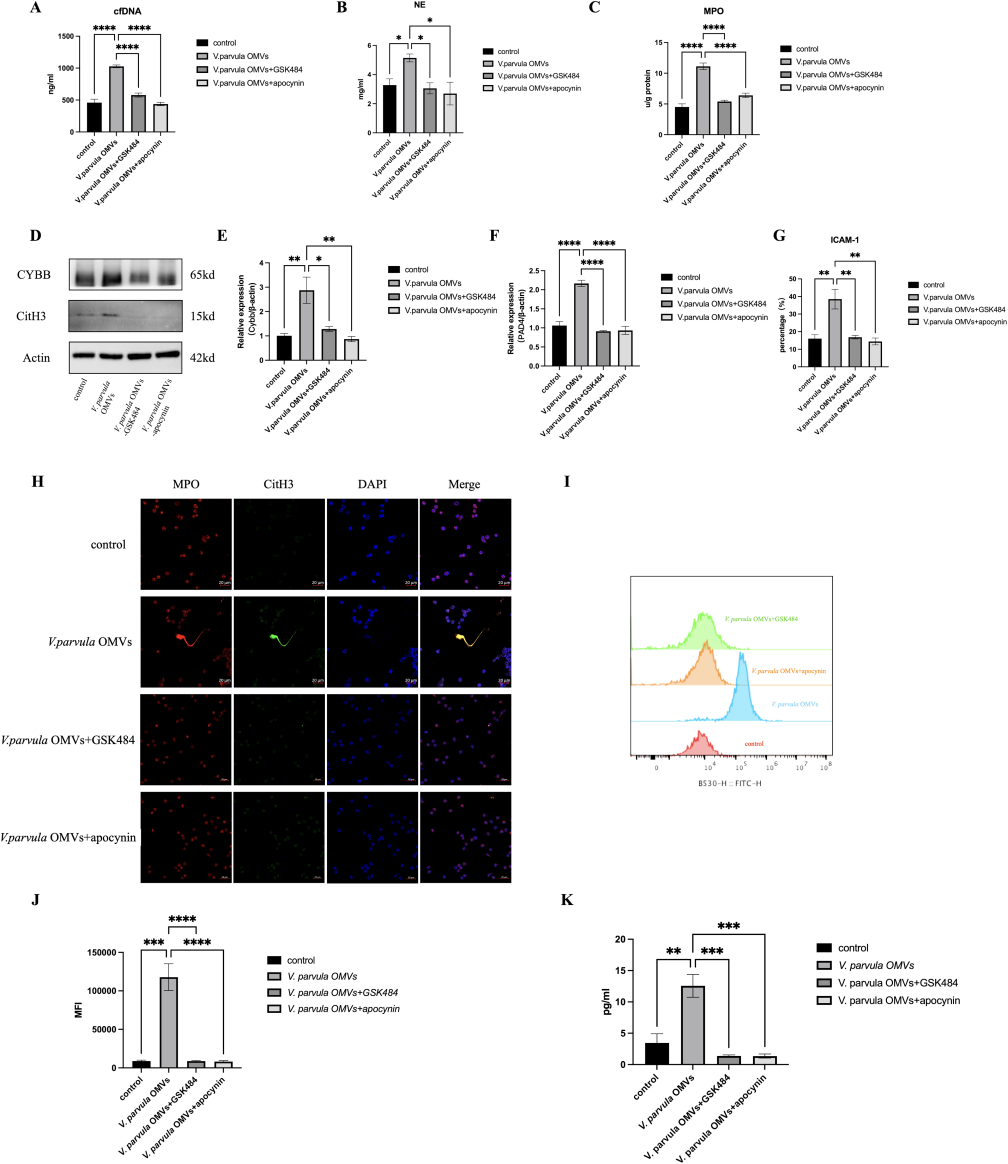
Neutrophil extracellular traps (NETs) formation after V. parvula OMVs stimulation. **(A)** Cell-free DNA (cf-DNA) expression in neutrophils. **(B)** Neutrophil elastase (NE) expression in neutrophils. **(C)** Myeloperoxidase (MPO) expression in neutrophils (*p < 0.05). **(D)** Protein levels of citrullinated histone H3 (CitH3) and CYBB in neutrophils. **(E)** Relative mRNA expression of *Cybb*. **(F)** Relative mRNA expression of *Pad4*. **(G)** Percentage of ICAM-1^+^ neutrophils by flow cytometry (FCM). **(H)** Immunofluorescence staining of NET formation in neutrophils (MPO, *red*; CitH3, *green*; DAPI, *blue*). *Scale bar*, 20 μm. **(I)** Histogram of reactive oxygen species (ROS) expression. **(J)** Mean fluorescence intensity (MFI) of ROS expression. **(K)** PAD4 expression in neutrophils. *p < 0.05, **p < 0.01, ***p < 0.001, ****p < 0.0001 (*n* = 3).

The formation of NETs in response to *V. parvula* OMVs was quantified by the cf-DNA, NE, and MPO expression. *V. parvula* OMVs led to enhanced cf-DNA production. The control group exhibited lower cf-DNA concentrations (465 ± 55.15 ng/ml) compared with the *V. parvula* OMV group (1,021 ± 45.12 ng/ml) (**Figure 5A**). The NE levels were also elevated in the *V. parvula* OMV group (5.145 ± 1.861 mg/ml) relative to the control group (3.285 ± 0.2256 mg/ml) (**Figure 5B**). The MPO expression was markedly increased in the *V. parvula* OMVs group (11.13 ± 2.788 U/g protein) compared with the control group (4.523 ± 1.880 U/g protein) (**Figure 5C**). The immunofluorescence (IF) assays corroborated these findings, showing enhanced CitH3 and MPO expression, indicative of the increased NET formation in response to *V. parvula* OMVs stimulation (**Figure 5H**).

### Effects of *V. parvula* OMVs on the ROS–PAD4 signaling pathway in ICAM-1^+^ neutrophils

The formation of NETs is closely related to the NADPH oxidase-derived ROS. **Figures 1I**, **1J** indicated that the *V. parvula* OMVs heightened the ROS production in active neutrophils. The Cybb gene encodes gp91-phox, the catalytic subunit of NADPH oxidase 2 (NOX2), which generates ROS. Notably, **Figures 1L**, **1M** showed that both mRNA and protein expression of CYBB were significantly increased in neutrophils following stimulation with *V. parvula* OMVs. Moreover, the elevated expression levels of the CYBB protein (**Figure 5D**) and the *Cybb* mRNA (**Figure 5E**) induced by *V. parvula* OMVs were attenuated by the CYBB inhibitor apocynin. NET formation, including the cf-DNA, NE, and MPO expression (**Figures 5A–C**), and the increased ICAM-1^+^ neutrophils (**Figure 5G**) induced by *V. parvula* OMVs were also attenuated in the *V. parvula* OMVs+apocynin group, suggesting that the effect of *V. parvula* OMVs on ICAM-1^+^ neutrophils was mediated by ROS production. In addition, the higher ROS level in the *V. parvula* OMVs group substantially declined in the *V. parvula* OMVs+apocynin group (*p* < 0.001; **Figures 5I, J**).

To verify the NET formation effect of the *V. parvula* OMVs on ICAM-1^+^ neutrophils, the NET inhibitor GSK484 was used. This resulted in a significant decrease of the cf-DNA, NE, and MPO expression in the *V. parvula* OMVs+GSK484 group compared with the *V. parvula* OMVs group (**Figures 5A–C**). The neutrophils in the *V. parvula* OMVs+GSK484 group demonstrated no elevated levels of the CitH3 protein expression compared with the control group (**Figure 5D**). Correspondingly, the unregulation of the *Pad4* mRNA expression in neutrophils was attenuated in the *V. parvula* OMVs+GSK484 group (**Figure 5F**). The IF assays corroborated these findings, showing that the increased NET formation was reduced in the *V. parvula* OMVs+GSK484 group (**Figure 5H**). Notably, the administration of GSK484 reversed the *V. parvula* OMVs-induced increase in ICAM-1^+^ neutrophils, effectively reducing the frequency of this cell population in the *V. parvula* OMVs+GSK484 group (**Figure 5G**). Simultaneously, the expression levels of the CYBB protein (**Figure 5D**) and the *Cybb* mRNA (**Figure 5E**) induced by *V. parvula* OMVs were attenuated in the *V. parvula* OMVs+GSK484 group. The excessive ROS production in neutrophils after *V. parvula* OMVs stimulation was attenuated in the *V. parvula* OMVs+GSK484 and *V. parvula* OMVs+apocynin groups (**Figures 5I, J**). In addition, there was a significant decrease in the expression of PAD4 in the *V. parvula* OMVs+GSK484 and *V. parvula* OMVs+apocynin groups compared with the *V. parvula* OMVs group (**Figure 5K**). These results suggest that the possible mechanism through which *V. parvula* OMVs increase ICAM-1^+^ neutrophils along with elevated NET formation was mainly through the ROS–PAD4 signaling pathway.

## Discussion

This study primarily unveiled the interaction between *V. parvula* OMVs and neutrophils by demonstrating phenotype and function. It was discovered that *V. parvula* OMVs altered the migration and apoptosis of neutrophils and the frequency of ICAM-1^+^ neutrophils. It was also revealed that the *V. parvula* OMV-induced ICAM^+^ neutrophils improved NET formation through the ROS–PAD4 signaling pathway. Hence, for the first time, we propose that the *V. parvula* OMV-induced ICAM-1^+^ neutrophils initiated NET formation to exaggerate the inflammation and injury to periodontal tissue.

*V. parvula* is an important early colonizer of the dental biofilm, promoting multispecies development and playing an indispensable role in community member metabolism (Rojas-Tapias et al., 2022). *V. parvula* OMVs contain LPS, lipids, and periplasmic proteins (Okamura et al., 2021). OMVs deliver pathogens to various locations, not only causing local infection directly but also activating host cells to induce cytokine/chemokine release (Gong et al., 2022).

It was demonstrated that *V. parvula* OMVs regulated the mRNA expression of *Icam-1* in neutrophils, as shown by RNA sequencing. Neutrophil-induced cytokines/chemokines play an essential function in the development of bacterial inflammation (Boero et al., 2021). ICAM-1 (CD54) is a transmembrane glycoprotein of the immunoglobulin superfamily and is expressed on endothelial and epithelial cells, neutrophils, and other leukocytes subsets (Morsing et al., 2021; Du et al., 2022). For stimulation with LPS, the expression of ICAM-1 on human neutrophils *in vitro* was noticeably upregulated (Murao et al., 2020). Mice with bacterial infection can also boost ICAM-1^+^ neutrophils, which was associated with neutrophil reverse transendothelial cell migration (Ode et al., 2018). With regard to its physiological role, ICAM-1 has been associated with migrating neutrophils and linked to the increased generation of ROS (Zoulikha et al., 2022).

It was identified that the increased ICAM-1^+^ neutrophils improved the MPO levels in neutrophils after stimulation with *V. parvula* OMVs. As shown in the cf-DNA, NE, and MPO assays, the formation of NETs noticeably increased in the *V. parvula* OMV group. NET formation represents a new innate immunity mechanism related to the defense against bacteria, virus, and fungi attacks through killing their growth, preventing their spread, and establishing an immune response to protect the host (Hofbauer et al., 2020; Neeli et al., 2023). The results of the neutrophil apoptosis assay were consistent with the increased formation of NETs. Apoptosis and NETosis are extremely important neutrophil death forms in disease. The cells of an organism are capable of dying through a mechanism of programmed cell death known as apoptosis. The apoptotic death process, as opposed to the necrotic death process, maintains the membrane integrity to limit the release of harmful neutrophil content (Perez-Figueroa et al., 2021). The release of NETs occurs mainly through a process of cell death called NETosis. NETosis involves multiple sequential steps, including neutrophil nuclear and cytoplasmic granule membrane disruption, chromatin relaxation, chromatin interaction with granule proteins, and chromatin release from neutrophils (Dejas et al., 2023). In this work, both neutrophil apoptosis and NETosis occurred at the same time in the *V. parvula* OMV group.

It was shown that UV irradiation simultaneously induced both apoptosis and NETosis in neutrophils (Azzouz et al., 2018). Drugs such as belinostat and panobinostat had a biphasic effect on neutrophils, inducing increased NETosis and apoptosis, switching neutrophil death from NETosis to apoptosis to maintain homeostasis (Hamam and Palaniyar, 2019). As a result, it was demonstrated that NETosis could be increased after stimulation with *V. parvula* OMVs, and the increased apoptosis might be an outcome of the maintenance of cellular homeostasis.

PAD4 catalyzes the arginine in histones to citrulline residues as a peptidyl arginine deiminase (Chen et al., 2021). PAD4 activity is associated with decondensed DNA in NET formation processes dependent on CYBB-induced ROS. The PAD4 expression was demonstrated to increase significantly after *V. parvula* OMV stimulation. It was also found that the *V. parvula* OMVs improved the neutrophil ROS production. ROS, as effector molecules, play a role in regulating the processes of NET formation and autophagy via microbial infection resistance and signaling transfer (Zhan et al., 2023). ICAM-1^+^ neutrophils can enhance the effector functions through improving the ROS production. It has been demonstrated that CYBB-derived ROS function in NET formation as signaling molecules (Damascena et al., 2022). It was also shown that the increased frequencies of ICAM-1^+^ neutrophils and NET formation were significantly attenuated following use of the PAD4 or CYBB inhibitor.

The results suggest that ICAM-1^+^ neutrophils may affect the formation of NETs through the ROS–PAD4 signaling pathway. Future research exploring the effects of *V. parvula* OMVs *in vivo* and the subsequent relationship between ICAM-1^+^ neutrophils and NET formation would be a point of focus in revealing the mechanisms of periodontal diseases and discovering new therapeutic strategies.

## Conclusion

In summary, our results showed that *V. parvula* OMVs improved neutrophil migration and directly increased ICAM-1 expression. ICAM-1^+^ neutrophils increased NET formation via the ROS–PAD4 signaling pathway. There is great potential to explore the pathogenic factors of OMVs and discover other therapeutic directions for ICAM-1^+^ neutrophils in the treatment of chronic periodontitis.

## Data Availability

The original contributions presented in the study are included in the article/supplementary material. Further inquiries can be directed to the corresponding authors.
